# Distribution Characteristics of Ground Echo Amplitude and Recognition of Signal Grazing Angle

**DOI:** 10.3390/s21248315

**Published:** 2021-12-12

**Authors:** Guangwei Zhang, Ping Li, Guolin Li, Ruili Jia

**Affiliations:** Mechatronical Engineering, Beijing Institute of Technology, Beijing 100081, China; zhangweweiha@126.com (G.Z.); 7920161015@bit.edu.cn (G.L.); jrl@bit.edu.cn (R.J.)

**Keywords:** THz ground clutter, bispectrum, amplitude probability density function (PDF), deep belief network

## Abstract

With the continuous advancement of electronic technology, terahertz technology has gradually been applied on radar. Since short wavelength causes severe ground clutter, this paper studies the amplitude distribution statistical characteristics of the terahertz radar clutter based on the measured data, and provides technical support for the radar clutter suppression. Clutter distribution is the function of the radar glancing angle. In order to achieve targeted suppression, in this paper, selected axial integral bispectrum (selected AIB) feature is selected as deep belief network (DBN)input to complete the radar glancing angle recognition and the network structure, network training method, robustness are analyzed also. The ground clutter amplitude distribution can follow normal distribution at 0~45° grazing angles. The Weibull distribution and G0 distribution can describe the amplitude probability density function of ground clutter at grazing angles 85° and 65°. The recognition rate of different signal grazing angles can reach 91% on three different terrains. At the same time, the wide applicability of the selected AIB feature is verified. The analysis results of ground clutter amplitude characteristics play an important role in the suppression of radar ground clutter.

## 1. Introduction

The rapid development of radar technology has continuously improved the detectability of radar systems, but the environment has a profound impact on target detection [1,2,3]. The environmental interference of ground and sea clutter has always been a hot research issue. Research on the characteristics of radar clutter can be traced back to the 1950s. Kaplan first proposed a statistical method to study clutter [4]. Researches about clutter characteristics have always received extensive attention. The key issue of clutter research is how to accurately model the clutter amplitude probability density function. At present, researchers have proposed a variety of models based on topography, vegetation, water content, grazing angle, and signal frequency bands. There are lognormal model [5,6], Gauss Rayleigh model [7,8], symmetric steady-state model [9,10], Weibull model [7,11], K distribution model [5,12], G0 model [13,14]. There is little research on the characteristics of near-field clutter above 100 GHz, which provides a direction for the follow-up development.

High-order spectrum analysis is a cutting-edge research direction in signal processing. Among the high-order spectrum, the bispectrum obtained from the third-order correlation function is the most widely used. Two-dimensional bispectrum features are difficult to be further processed. The integral bispectrum transforms two-dimensional the bispectrum function into a one-dimensional function, which is conducive to the calculation. The radial integral bispectrum (RIB) [15], axial integral bispectrum (AIB) [16], circular integral bispectrum (CIB) [17], surrounding-line integrated bispectrum (SIB) [18] and other methods have been proposed. Professor Zhang Xianda proposed the selected bispectrum [19].

The deep belief network can be used for unsupervised learning and supervised learning. DBN is a probabilistic generative model with a traditional discriminant. The generative model establishes joint distribution between observation data and labels. By training the weights, the entire neural network can generate training labels according to the maximum probability [20].

The amplitude distribution characteristics of ground clutter are the basis of radar clutter suppression and play a key role in target detection. There is little research on the characteristics of ground clutter in the terahertz frequency band [21,22]. This paper is based on measured data to fill this gap. Previous studies mainly focused on targeted interference suppression of signals with large grazing angle (≥60°) signal. Since other glancing angles are not considered, no significant research results were obtained [23]. Previously, for grazing angle recognition, the backscattering coefficient of different terrains was mainly studied, but the backscattering coefficient is a multi-factor function. As the environment changes, the backscattering coefficient is also changing, which does not have wide applicability [24,25]. In this paper, measure THz ground clutter from alpine meadows, alpine dry grassland and alpine swamp and echo signal selected AIB are used as DBN network input to recognize grazing angle, to suppress clutter at different grazing angles. The bispectrum characteristics of ground clutter are studied. The amplitude distribution characteristics of ground echo at different grazing angles are analyzed. The axial integrated bispectrum, radial integrated bispectrum, circular integrated bispectrum, surrounding-line integrated bispectrum and selected axial integrated bispectrum (selected-AIB) are calculated. The deep belief network is used to identify different grazing angle echo.

The paper is organized as follows. The amplitude statistical distribution and bispectrum are recalled and analysed in Section 2. The selected AIB and network optimization are analysed in Section 3. The statistical characteristics of the amplitude are analysed based on the measured data in Section 4. DBN are used to classify and identify grazing angles in Section 4. The research content is summarized in Section 5.

## 2. Area Target Scattering Model and Bispectrum

### 2.1. Area Target Scattering Model

The terahertz signal wavelength is far smaller than the size of the area target. According to the electromagnetic scattering theory, the scattering characteristics of the area target already belong to the quasi-optical zone. The interaction between the various parts of the scatterer is very weak, and the scattering is almost a local phenomenon rather than accumulation process. The area integration current comes from the stagnation points and the integration end points. The scattering energy of the area target mainly comes from these scattering points, which are called the scattering center of the area target. The echo characteristics of the area target are determined by multiple scattering centers.

The Geometric Theory Diffraction (GTD) [26] is a classic theory for describing scattering center. The approximate formula of the scattering field can be described as follows:

(1) $$E_{q}(f(t),\theta ,\gamma )=\sum_{m=1}^{M}A_{m}(t)S_{qm}(\theta ,\gamma ){\left(\frac{f}{f_{0}}\right)}^{a_{m}}e^{-j4\pi f(x_{m}\cos\gamma \cos\theta +y_{m}\cos\gamma \sin\theta +z_{m}\sin\gamma )/c}$$

The signal energy mainly comes fromstrong scattering center. m is the serial number of the scattering center. $f_{0}$ is the center frequency of the broadband signal, q is specific polarization combination of transmit and receive. (x,y,z),  θ,  γ describe the position of the scattering center and the attitude angle. $S_{qm}$ is the scattering coefficient factor. $a_{m}$ is central parameter. f is signal radio frequency. c is speed of light.

**Figure 1:**
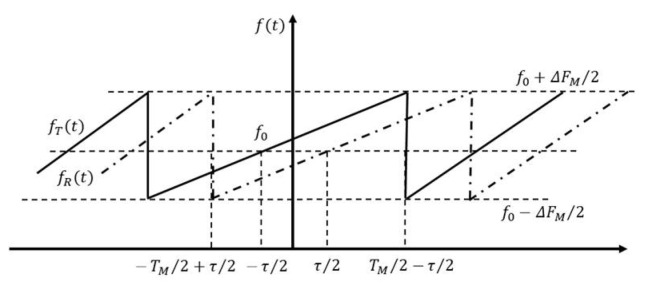
Sawtooth FM frequency time relationship.

The total scattered field of the area target can be simplified as:

(2) $$E_{q}(f(t))={\sum_{m=1}^{M}A_{m}(t)S_{qm}(\frac{f}{f_{0}})}^{a_{m}}e^{-j4\pi fr_{m}/c}$$

where, $r_{m}$ represents the distance from the scattering center to the radar in the direction of the radar’s sight line. (ff0)am reflects the relationship between the scattering intensity of the scattering center and the frequency. The area target scattering can be expressed by the above formula. In order to facilitate the analysis and simplify the mathematical model of the signal, the noise term is omitted, and the signal is considered as a sawtooth chirp signal. Sawtooth FM frequency time relationship is shown in Figure 1.

The transmitted signal frequency is expressed as:

(3) $$f_{T}(t)=\Delta F_{M}f_{M}t+\frac{1}{2}\Delta F_{M}f_{M}\tau +f_{0}$$

The received signal frequency is expressed as:

(4) $$f_{R}(t)=\Delta F_{M}f_{M}t-\frac{1}{2}\Delta F_{M}f_{M}\tau +f_{0}$$

$∆F_{M}$ is the frequency offset. τ is the delay time. $f_{M}$ is modulation frequency.

The ground echo can be expressed as:

(5) $$x_{q}(t)=E_{q}(f_{R}(t))={\sum_{m=1}^{M}A_{m}(t)S_{qm}e^{-j4\pi f_{0}/c}(1+\frac{\Delta F_{M}f_{M}t-\frac{1}{2}\Delta F_{M}f_{M}\tau }{f_{0}})}^{a_{m}}e^{-j4\pi f_{R}(t)r_{m}/c}$$

(6) $$r_{m}=R_{0m}+v_{0m}t$$

$R_{0m},\;v_{0m}$ are the initial distance and velocity of scattering center (considering the case of uniform velocity).

(7) $$a_{qm}=A_{m}(t)S_{qm}e^{-j4\pi f_{0}/c}$$

(8) $$\omega_{m}=-j4\pi f_{R}(t)r_{m}/c$$

For terahertz radar, when the bandwidth is the same, it has smaller relative bandwidth. The above formula can be further simplified as:

(9) $$x(t)=\sum_{m=1}^{M}a_{qm}e^{jt\omega_{m}}$$

For the sawtooth chirp system, the echo from area target multiple scattering points is a multiple sinusoidal signals combined model.

Substitute $a_{qm},\;\omega_{m}$ into the formula:

(10) $$x(t)=\sum_{m=1}^{M}A_{m}(t)S_{qm}\exp\{-j4\pi (f_{R}(t))\frac{r_{m}}{c}\}$$

(11) $$\begin{array}{l} x(t)=\sum_{m=1}^{M}A_{m}(t)S_{qm}\exp\{-j4\pi (\Delta F_{M}f_{M}t-\Delta F_{M}f_{M}(\frac{R_{0m}+v_{0m}t}{c})+f_{0})\frac{R_{0m}+v_{0m}t}{c}\}\\ \begin{array}{cc} & =\sum_{m=1}^{M}A_{m}(t)S_{qm}\exp\{-j4\pi [\frac{v_{0m}\Delta F_{M}f_{M}(2c-v_{0m})}{2c^{2}}t^{2}+\left(\frac{R_{0m}\Delta F_{M}f_{M}(2c-v_{0m})+2cv_{0m}f_{0}-\Delta F_{M}f_{M}R_{0m}v_{0m}}{2c^{2}}\right)t+\frac{2cR_{0m}f_{0}-\Delta F_{M}f_{M}R_{0m}{}^{2}}{2c^{2}}]\} \end{array} \end{array}$$

The ground echo received by the radar is the vector sum of the backscattered electromagnetic field. The center of the ground scatterer in the radar beam changes with space and time, which will affect the clutter amplitude. Therefore, the description for the amplitude distribution characteristics requires statistical methods.

So far, many echo models have been proposed. The more commonly recognized amplitude probability density distribution function models include the Rayleigh distribution, lognormal distribution, Weibull distribution, K distribution, etc. In recent years, the gamma distribution, and G0 distribution have been proposed.

#### 2.1.1. Rayleigh Distribution

Within the radar resolution range, if the number of scatterers is large, the echo amplitude and phase have random characteristics. In the case of low-resolution radar, Rayleigh distribution is more suitable for describing ground clutter distribution. The echo amplitude follows the Rayleigh distribution. If x is the envelope amplitude, and $\sigma^{2}$ is power, the probability density function is:

(12) $$f(x)=\frac{x}{\sigma^{2}}\exp\left(-\frac{x^{2}}{2\sigma^{2}}\right)\begin{array}{ccc} & & x\geq 0 \end{array}$$

#### 2.1.2. Lognormal Distribution

x is the envelope amplitude of the clutter. The probability density function is:

(13) $$f(x)=\frac{1}{\sqrt{2\pi }\sigma x}\exp\left(-\frac{{\left(\ln x-\mu \right)}^{2}}{2\sigma^{2}}\right)\begin{array}{ccc} & & x\geq 0 \end{array}$$

μ —Scale parameter, which represents the median of the distribution.

σ —The shape parameter, which represents the skewness of the distribution.

#### 2.1.3. Weibull Distribution

In the case of close detection, that is, severe clutter, the Weibull distribution is more suitable for describing ground clutter distribution. The asymmetry of this distribution is weaker than lognormal distribution. x is the envelope amplitude. The probability density function is:

(14) $$f(x)=\frac{p}{q}{\left(\frac{x}{q}\right)}^{p-1}\exp\left[-{\left(\frac{x}{q}\right)}^{p}\right]x\geq 0$$

q—Scale parameter, which represents the median of the distribution.

p—Shape parameter, which represents the skewness of the distribution.

#### 2.1.4. K Distribution

The clutter distribution models of the Rayleigh distribution, lognormal distribution and Weibull distribution are all based on single point statistics, so they are only suitable for single pulse detection. Their main disadvantage is the lack of time and space correlation. In recent years, the K-distribution mixing model has been introduced.

(15) $$f_{k}(x)=\frac{2}{a\Gamma (v+1)}{(\frac{x}{2a})}^{v+1}K_{v}(\frac{x}{a})\begin{array}{cc} & x\geq 0 \end{array}$$

where x is the amplitude and a(a>0) is the quantization parameter whose change determines the characteristics of the clutter distribution. v is the shape parameter −1<v<∞, and $K_{v}$ is the modified Bessel function.

#### 2.1.5. Gamma Distribution

The gamma distribution clutter model probability density function is:

(16) $$f(x,\mu ,L)=\frac{L}{\mu \Gamma (L)}{\left(\frac{Lx}{\mu }\right)}^{L-1}\exp(-\frac{Lx}{\mu })\begin{array}{cc} & x\geq 0 \end{array}$$

μ is the scale parameter, L is the shape parameter.

G0 distribution.

The G0 distribution clutter model probability density function is:

(17) $$f(x,L,\gamma ,\alpha )=\frac{2L^{L}\Gamma (L-\alpha )}{\gamma^{\alpha }\Gamma (L)\Gamma (-\alpha )}\frac{x^{2L-1}}{{\left(\gamma +Lx^{2}\right)}^{L-a}}\begin{array}{cc} & x\geq 0 \end{array}$$

L(L>0) is the equivalent sight and γ(γ>0) is the scale parameter, which is related to the average value of the scattered energy. α(α<0) is the shape parameter, which reflects the uniformity of the observation area.

K−root distribution.

The K−root distribution clutter model probability density function is:

(18) $$f(x,L,v,\mu )=\frac{2}{x}{\left(\frac{Lvx}{\mu }\right)}^{\frac{L+v}{2}}\frac{L}{\Gamma (L)\Gamma (v)}K_{V-L}(2\sqrt{\frac{Lvx}{\mu }})\begin{array}{cc} & x\geq 0 \end{array}$$

where $K_{V-L}\left(\cdot \right)$ is the second modified Bessel function and Γ(·) is the gamma function. The K-root model can be regarded as a product model of two independent random variables. One variable follows the gamma distribution with mean of 1 and shape parameter L, and the other variable follows the gamma distribution with mean μ and shape parameter v. Gamma distribution, G0 distribution and k-root distribution are more flexible to describe changes in clutter distribution.

### 2.2. Bispectrum Characteristic

The bispectrum of continuous-time signal x(t) is defined as:

(19) $$\begin{array}{l} B(\omega_{1},\omega_{2})=S_{3x}(\omega_{1},\omega_{2})=\sum_{\tau_{1}=-\infty }^{\infty }\sum_{\tau_{2}=-\infty }^{\infty }\left|C_{3x}(\tau_{1},\tau_{2})\right|\exp[-i(\omega_{1}\tau_{1}+\omega_{2}\tau_{2})] \end{array}$$

where

(20) $$\begin{array}{l} C_{3x}(\tau_{1},\tau_{2})=\int_{-\infty }^{\infty }x^{*}(t)x(t+\tau_{1})x(t+\tau_{2})dt\\ \begin{array}{ccc} & & \begin{array}{cc} & =E\left\{x^{*}(t)x(t+\tau_{1})x(t+\tau_{2})\right\} \end{array} \end{array} \end{array}$$

The bispectrum is uniquely defined by its values in the triangular region $0\leq \omega_{2}\leq \omega_{1}\leq \omega_{2}+\omega_{1}\leq \pi$. The bispectrum can not only reflect the signal amplitude information but also reflect the phase information. The bispectrum can handle a variety of signal models, such as non-Gaussian, nonlinear, noncausal, nonminimum phase, Gaussian coloured noise and blind signals. In theory, the interference of Gaussian noise is completely suppressed, because the third-order cumulant of one-dimensional Gaussian stationary random signals is zero.

## 3. Selected AIB and Network Optimization

Some feature points are lost in the RIB calculation process, and these points contain useful information. The AIB method also has disadvantage, and most of the integral bispectrum phase information are lost. $B_{p}\left(a,\theta \right)$ is the polar coordinate representation of $B\left(\omega_{1},\omega_{2}\right)$ in the CIB. Note that, $B_{p}\left(a,k\pi /2\right)$ with integer k provides no phase information. a is near kπ/2, and $B_{p}\left(a,\theta \right)$ provides a little phase information. Only partial phase and amplitude information features are used in the SIB, and the features are not fully utilized.

The axial integral bispectrum (AIB):

(21) $$AIB=\frac{1}{2\pi }\int_{-\infty }^{\infty }B(\omega_{1},\omega_{2})d\omega_{2}=\frac{1}{2\pi }\int_{-\infty }^{\infty }B(\omega_{1},\omega_{2})d\omega_{1}$$

The radial integral bispectrum (RIB):

(22) $$RIB(a)=I(a)=\int_{0}^{1/(1+a)}B(f_{1},af_{1})df_{1}$$

The circular integral bispectrum (CIB):

(23) $$CIB(a)=\int_{0}^{2\pi }B_{p}(a,\theta )d\theta$$

The surrounding-line integrated bispectrum (SIB):

(24) $$SIB(s)=\sum_{R}B(\omega_{1},\omega_{2})$$

### 3.1. Selected AIB

A one-dimensional Fisher class [19] is introduced to calculate the signal AIB feature point discriminant degree. The AIB feature points with the high discriminant are selected and re-taken as the new feature vectors. The trivial AIB feature points in the signal are deleted. The dimension of the feature vector is reduced so that the network training time gets shorter, and the application ability of the network is improved.k={1,2,⋯,512}. The one-dimensional Fisher is expressed as:

(25) $$Xm^{\left(ij\right)}(a)=\frac{\sum_{l=i,j}{\left[mean_{k}(x_{k}{}^{\left(i\right)}(a))-mean_{l}(mean_{k}(x_{k}{}^{\left(i\right)}(a)))\right]}^{2}}{\sum_{l=i,j}{\mathrm{var}}_{k}(x_{k}{}^{\left(i\right)}(a))}$$

$mean_{k}\left(x_{k}^{\left(i\right)}\left(a\right)\right)$ is the mean (centroid) of all the AIBs in the ith class.

$var_{k}\left(x_{k}^{\left(i\right)}\left(a\right)\right)$ is the variance of all the AIBs in the ith class.

$mean_{l}\left(mean_{k}\left(x_{k}^{\left(i\right)}\left(a\right)\right)\right)$ is the total centroid of all the sample AIBs in all the classes.

$Xm^{\left(ij\right)}\left(a\right)$ indicates the discriminant between the ith class and jth class at the a point. The four grazing angles are needed to calculate $m^{12}\left(a\right)$, $m^{13}\left(a\right)$, $m^{14}\left(a\right)$, $m^{23}\left(a\right)$, $m^{24}\left(a\right)$, and $m^{34}\left(a\right)$.

The feature extraction process:

Third-order cumulant is used the to calculate the signal bispectrum and calculate the signal AIB feature according to the bispectrum. Calculate the discriminant degree of different AIB features points according to [25] and perform standardization processing.

(26) $${\bar{m}}^{\left(ij\right)}(a_{n})=\frac{m^{\left(ij\right)}(a_{n})}{\sqrt{\sum_{K=1}^{M}{\left[m^{\left(ij\right)}(a_{n})\right]}^{2}}}$$

The first Q points are taked as signal feature vectors.$m^{\left(ij\right)}\left(a_{1}\right)>m^{\left(ij\right)}\left(a_{2}\right)\cdots >m^{\left(ij\right)}\left(a_{Q}\right)$. Re-establish the four grazing angle signal feature vectors. The Q value needs to be adjusted. i and j class selected AIB feature vectors are:

i class selected -AIB:$\left\{s_{i}\left(a_{1}\right),s_{i}\left(a_{2}\right),\cdots ,s_{i}\left(a_{Q}\right)\right\}$.

j class selected -AIB: $\left\{s_{j}\left(a_{1}\right),s_{j}\left(a_{2}\right),\cdots ,s_{j}\left(a_{Q}\right)\right\}$.

The selected-AIB is used as the DBN input to train the network and output the prediction result.

### 3.2. Data Preprocessing

During preprocessing, one-dimensional Fisher is used to extract and amplify important features, while trivial features are deleted and suppressed. The data are normalized to improve the convergence speed of the model and prevent the model gradient exploding. The data are randomly scrambled, and K-fold cross validation is performed so that the data sequence does not affect network training. The advantage is to increase the randomness to avoid regular data deviation or falling into local optimum, which improves the generalization performance of the network.

### 3.3. Optimization of Deep Belief Network

**Figure 2:**
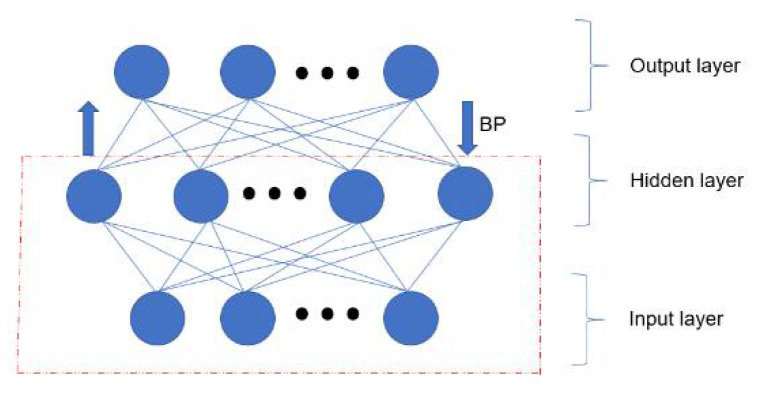
DBN network.

Considering the training speed of the network, the three-layer network is selected for classification. The second hidden layer activation function is restricted Boltzmann machine (RBM). When the feature vector is mapped to different feature spaces, it retains as much feature information as possible to establish joint distribution between the observation data and the label. The number of hidden units is used as a parameter to be adjusted. The third layer is the output layer, which is a fully connected layer. DBN network is shown in Figure 2.

$V=\left[v_{1},v_{2},\cdots ,v_{N}\right]$ is visible layer state. $A=\left[a_{1},a_{2},\cdots ,a_{N}\right]$ is the offset of the visible layer. $H=\left[h_{1},h_{2},\cdots ,h_{M}\right]$ is state of the hidden layer. $B=\left[b_{1},b_{2},\cdots ,b_{M}\right]$ is bias of the hidden layer.${\left[W_{ij}\right]}_{NM}$ is weight matrix between the visible layer and the hidden layer. The visible layer is real-valued, and the potential function can be expressed as:

(27) $$E(V,H)=\sum_{i=1\cdots N}^{}\frac{1}{2\sigma_{i}^{2}}{\left(v_{i}-a_{i}\right)}^{2}-VWH^{T}-HB^{T}$$

$\sigma_{i}$ is the standard deviation of the neuron in the visible layer. By defining the energy function, the probability distribution function can be defined:

(28) $$P(V)=\frac{\sum_{H}\exp(-E(V,H))}{\sum_{Y}\sum_{H}\exp(-E(V,H))}$$

It has been proven that RBM can approximate probability distribution functions arbitrarily. Use the maximum likelihood estimation method to describe the input data probability distribution function. In order to simplify the calculation, likelihood function that minimizes the negative is adopted:

(29) $$Loss(\theta )=-\sum_{i=1}\log p(x_{i}|\theta )$$

θ={W,A,B} is the parameter that needs to be adjusted during training. The training process of the network is the optimization process of parameter θ.

Further processing of the above potential function, get the two important inferences P(H|V), P(V|H).P(H|V) indicates the ability of the data features extracted through the network to represent the original data, and judges whether the extracted hidden features can be used for classification. P(V|H) is the basis of network optimization and adjustment, and provides adjustment directions, and guarantees the smooth progress of the entire network training. According to the visible layer, the state of all neurons are directly calculated. This parallel mechanism can greatly speed up training and inference. The reason why it can be calculated in parallel is that RBM eliminates the dependence of neurons between layers.

The hidden layer training process:

(30) $$p(H={\left[1,\ldots ,1\right]}_{1\times M}|V)=\prod_{j=1}^{M}\frac{1}{1+\exp(-VW-B)}$$

The visible layer training process:

(31) $$p(V={\left[1,\ldots ,1\right]}_{1\times N}|H)=\prod_{i=1}^{N}\frac{1}{1+\exp(-HW^{T}-A)}$$

The training process of the network is actually to search the optimal parameters, and the optimal parameters are:

(32) $$\theta^{*}=\arg\min loss(\theta )$$

Use the gradient descent method to update the parameters:

(33) $$\theta (t+1)=\theta (t)-\eta \frac{\partial Loss(\theta )}{\partial \theta }$$

η represents the learning rate, which is used to control the speed of learning. Aiming at the Bernoulli restricted Boltzmann machine, after adopting contrastive divergence (CD-K)approximation calculation, various parameters are obtained:

(34) $$\frac{\partial }{\partial W}Loss(\theta )=V_{k}H_{k}-V_{0}H_{0}$$

(35) $$\frac{\partial }{\partial A}Loss(\theta )=V_{k}-V_{0}$$

(36) $$\frac{\partial }{\partial B}Loss(\theta )=H_{k}-H_{0}$$

The training process of DBN can be regarded as the initialization of weight parameters, which overcomes the shortcomings of BP network. Back Propagation-Deep Belief Network (BP-DBN) needs to only perform partial search on the weight parameter space. Compared with forward neural network, BP-DBN convergence time is faster.

## 4. Feature Extraction and Network Optimization

### 4.1. Measurement and Test Radar

**Figure 3:**
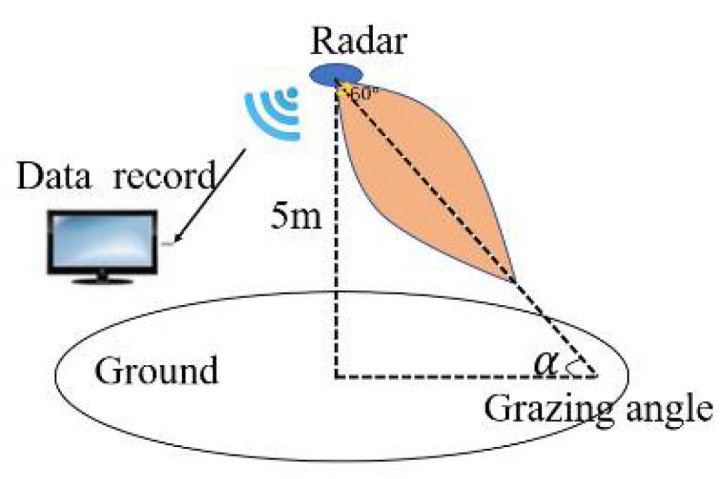
Test equipment.

**Figure 4:**
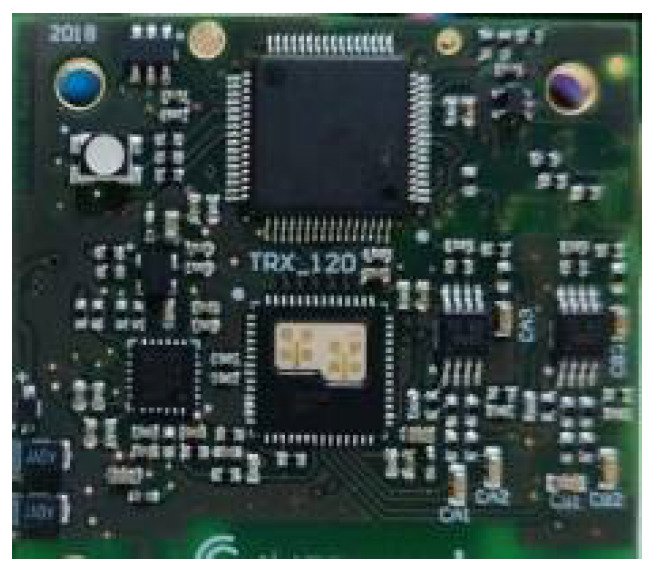
Integrated test radar.

The test signal frequency is 120 GHz, and the bandwidth is 1 GHz, and the modulation method is the sawtooth chirp signal. Radar is an integrated transceiver system. Transmitting power is 1 mW. The angle between the centerline of the beam and the ground is defined as the glancing angle. The grazing angle is controlled by the rotatable pan-tilt platform. The beam width is 60°. Modulation period is 946 µs. The sampling rate of the beat frequency signal is 1 MHz. There are 512 points per period. The test schematic is shown in Figure 3. The wavelength is 2.5 mm. The antenna is array antenna, as shown in Figure 4. It features on-chip MMW built-in-self-test (BIST) circuits, a harmonic transceiver, software linearization (SWL) circuits, and a digital interface. This chipset has been tested in a low-cost package, where the antennas are integrated. The vertical height of the test equipment from the ground is 5 m.

**Figure 5:**
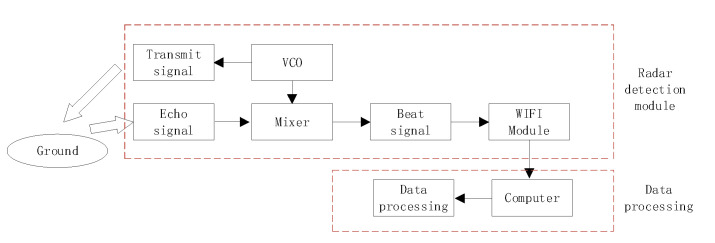
Data processing flow.

The radar transmits 120 GHz signal. The echo reflects from the ground when it touches the ground. After the radar receives the echo, it performs mixing frequency processing and outputs beat frequency signal. The computer is connected to the radar data output terminal to record data. Data processing flow is shown in Figure 5. First, analyze the ground clutter amplitude probability density distribution. The ground clutter amplitude PDF is compared with the normal distribution, Rayleigh distribution, K distribution, Weibull distribution, lognormal distribution, gamma distribution, G0 distribution, and K-root distribution.

### 4.2. Alpine Meadow Amplitude PDF

**Figure 6:**
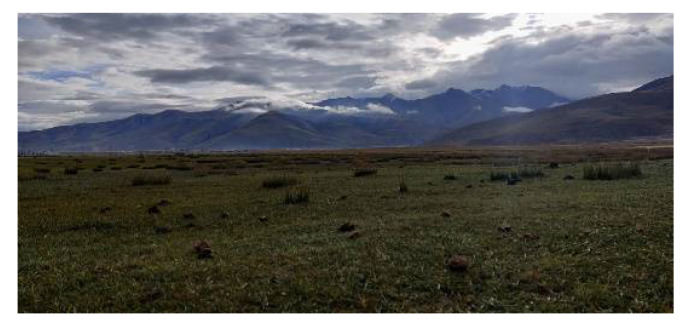
Alpine meadow test environment.

The alpine meadow is covered with low vegetation, and the soil is moist. The ground is relatively flat. An alpine meadow test environment is shown in Figure 6.

The 200 periodic signals are used to analyse signal amplitude PDF. The per period contains 512 points. Range between maximum amplitude and minimum amplitude is divided into 512 parts. The point number in per part is calculated, and know the measured signal PDF curve. According to the theoretical distribution formulas, theoretical distribution curves can be obtained.

The measured data amplitude PDF contains 512 points. Theoretical distribution curves also contains 512 points. The corresponding points are subtracted, and then difference sums is used as the fitting error. Use fitting error to determine the fit degree between the measured signal PDF curve and theoretical distribution curves.

s(i) is the measured data amplitude PDF curve. k(i) is theoretical distributions curve. The fitting error is expressed as:

(37) $$err=\sum_{i=1}^{512}\left|s(i)-k(i)\right|$$

The goodness of fit is introduced to describe the shape fit degree between the theoretical distribution curve and the ground echo amplitude PDF curve. The statistical parameter of goodness is expressed as the determination coefficient $R^{2}$. The maximum value $R^{2}$ is 1, indicating that the shape fit result between the curves is perfect.


(38) $$R^{2}=1-\frac{\sum_{i=1}^{512}{\left(y_{i}-Y_{i}\right)}^{2}}{\sum_{i=1}^{512}{\left|Y_{i}-\bar{Y}\right|}^{2}}$$

**Figure 7:**
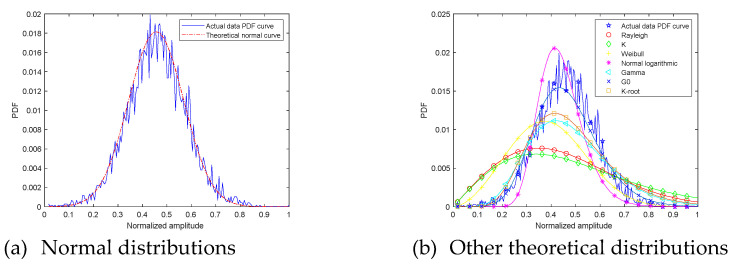
The 20° ground clutter amplitude PDF and theoretical curves.

Next, the measured echo amplitude PDF curve is compared with theoretical distributions curve. At grazing angle 20°, the ground echo amplitude PDF and theoretical distributions curve are shown in Figure 7. The fitting error and goodness of fit $R^{2}$ are shown in Table 1.

The first picture is the comparison between the measured data amplitude distribution and the normal distribution. The second picture shows the comparison between the measured data amplitude distribution and other theoretical distributions. Different theoretical distribution curves are distinguished by different curve shapes. Through observation, it is found that the curves degree of fit between the measured data amplitude distribution and the normal distribution is high. Other theoretical amplitude distribution curves and measured data distribution curves reflect the differences in peak, peak position, and curve fluctuations.

Fitting error describes the difference from the digital perspective. Fitting error from measured signal amplitude PDF curve and normal distribution curve is 0.13. It is the smallest among all fitting errors. Fitting error from measured signal amplitude PDF curve and G0 distribution curve is 0.18. $R^{2}$ describes the difference from the curve shape perspective. Normal distribution curve $R^{2}$ is 0.97, and it can explain that normal distribution curve shape is closest to measured signal amplitude PDF curve. The suboptimal $R^{2}$ value is G0 0.95. Through the above analysis, at grazing angle 20°, measured signal amplitude PDF curve can be described as normal distribution.

**Figure 8:**
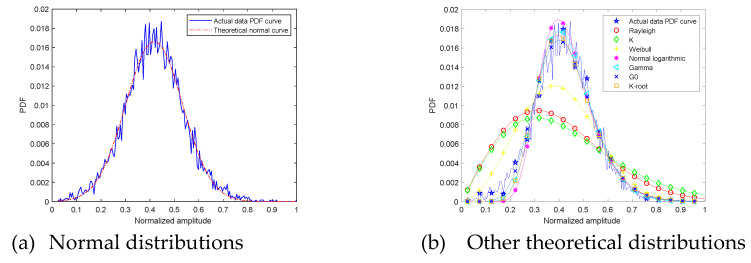
The 45° ground clutter amplitude PDF and theoretical curves.

At grazing angle 45°, the ground echo amplitude PDF and theoretical distributions curve are shown in Figure 8. The fitting error and goodness of fit $R^{2}$ are shown in Table 2.

The first picture is the comparison between the measured data amplitude distribution and the normal distribution. The second picture shows the comparison between the measured data amplitude distribution and other theoretical distributions. Through observation, it is found that the degree of fit between the measured data amplitude distribution and the normal distribution is high at grazing angle 45°. The four distribution functions of Lognormal, Gamma, G0, K-root four distribution functions gradually approach the measured data amplitude distribution, and the divergence gradually decreases.

Fitting error from measured signal amplitude PDF curve and normal distribution curve is 0.10. It is the smallest among all fitting errors. Fitting error from measured signal amplitude PDF curve and G0 distribution curve is 0.13. Normal distribution and measured signal amplitude PDF curve $R^{2}$ is 0.98, and it explain that normal distribution curve shape is closest to measured signal amplitude PDF curve. The suboptimal $R^{2}$ value is G0 0.97. Through the above analysis, at grazing angle 45°, measured signal amplitude PDF curve can be described also as normal distribution.

**Figure 9:**
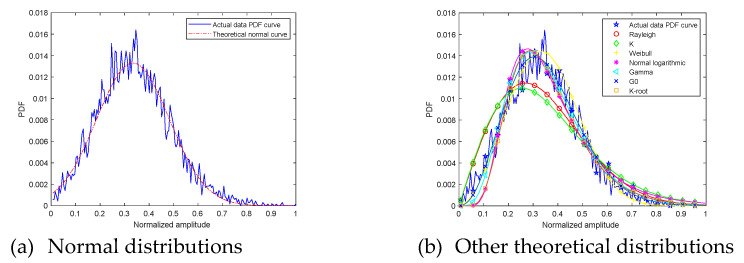
The 65° ground clutter PDF and theoretical curves.

At grazing angle 65°, the ground echo amplitude PDF and theoretical distributions curve are shown in Figure 9. The fitting error and goodness of fit $R^{2}$ are shown in Table 3.

The first picture is the comparison between the measured data amplitude distribution and the normal distribution. It is obvious that the peak value position of the measured data amplitude distribution curve moves forward with the increase of the grazing angle. The normal distribution is no longer the optimal description curve. Through observation, it is found that the degree of fit between the measured data amplitude distribution and the G0 distribution curve distribution is high.

Fitting error from measured signal amplitude PDF curve and normal distribution curve is 0.15. Fitting error from measured signal amplitude PDF curve and G0 distribution curve is 0.14, same as Weibull distribution. It is the smallest among all fitting errors. Normal distribution curve $R^{2}$ is 0.94. Weibull distribution and G0 distribution curve $R^{2}$ value is G0 0.96. Through the above analysis, at grazing angle 65°. In terms of $R^{2}$ and fitting error, Weibull distribution and G0 distribution has ability to describe the measured signal amplitude PDF curve.

**Figure 10:**
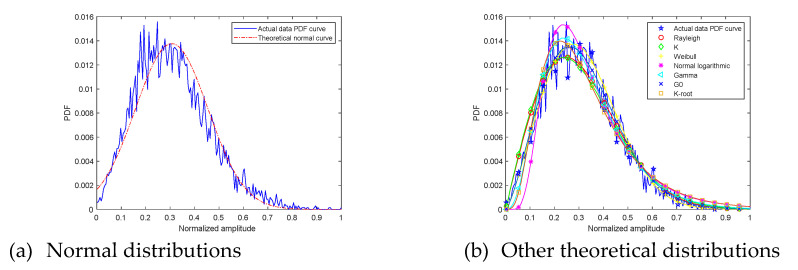
The 85° real ground clutter amplitude PDF and theoretical curves.

At grazing angle 85°, the ground echo amplitude PDF and theoretical distributions curve are shown in Figure 10. The fitting error and goodness of fit $R^{2}$ are shown in Table 4.

It is observed that the peak value position of the measured data distributions curve further moves forward, and the difference with the normal distribution curve is further enlarged. Other theoretical distribution curves are gradually approaching the measured data distribution curve. Weibull distribution and G0 distribution from peak value, peak position, and curve fluctuation can describe the measurement data distribution well.

Fitting error from measured signal amplitude PDF curve and normal distribution curve is 0.14. Fitting error from measured signal amplitude PDF curve and G0 distribution curve is 0.12. Weibull Fitting error is 0.12. From the digital perspective, G0 distribution curve and Weibull distribution curve are optimal description curve. Normal distribution curve $R^{2}$ is 0.94. G0 distribution curve $R^{2}$ is 0.97. Weibull distribution curve $R^{2}$ is 0.96 and is the basically same as G0 distribution $R^{2}$. Weibull distribution and G0 distribution curve are close to measured signal amplitude PDF curve, at grazing angle 85°.

## 5. DBN Classification

**Figure 11:**
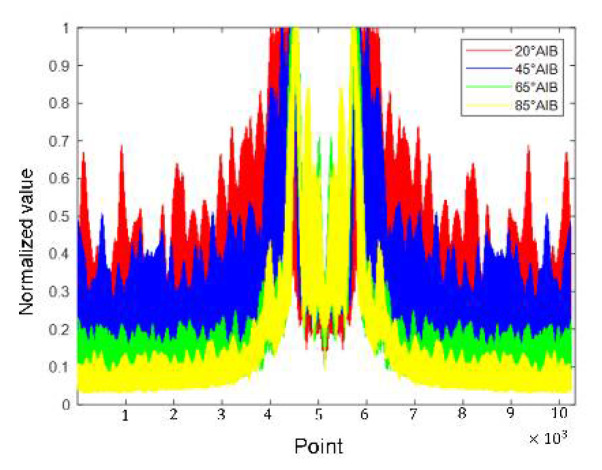
Alpine meadow echo AIB.

**Figure 12:**
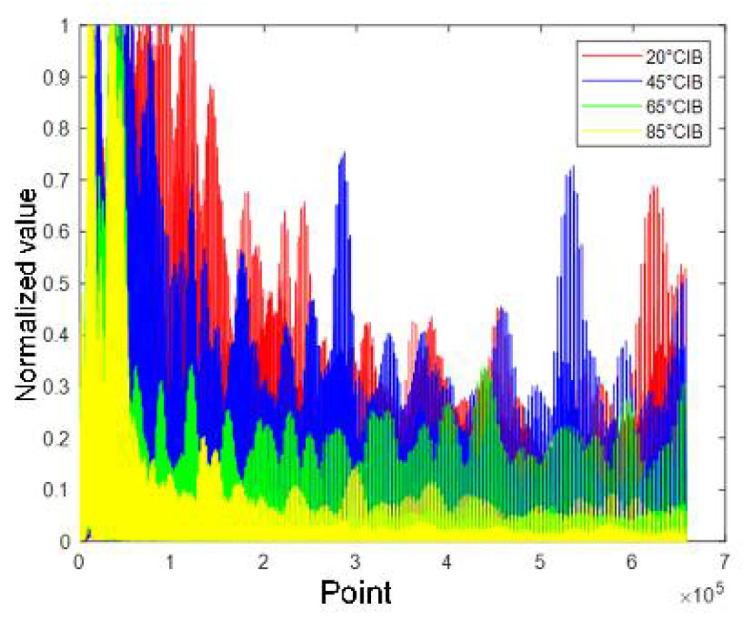
Alpine meadow echo CIB.

**Figure 13:**
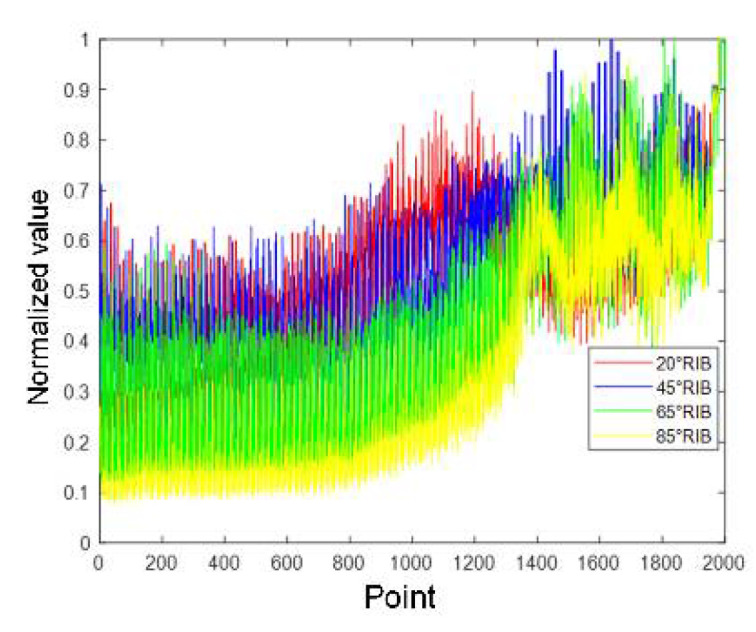
Alpine meadow echo RIB.

**Figure 14:**
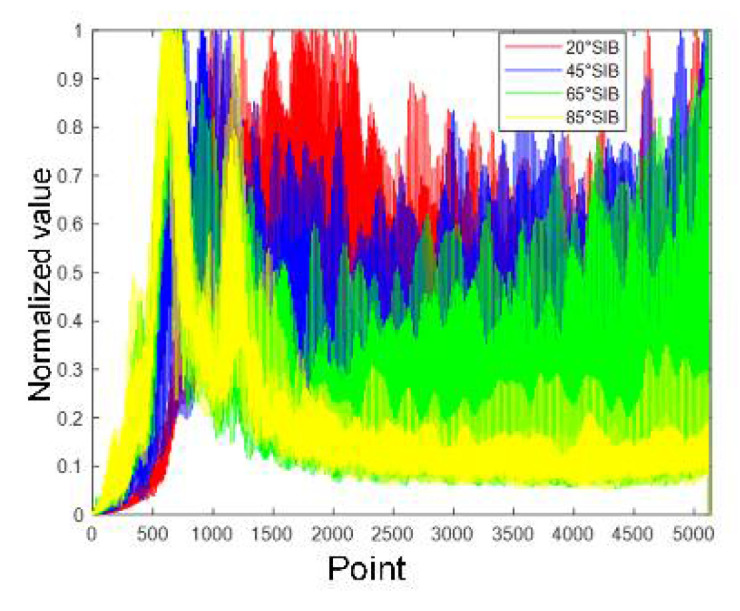
Alpine meadow echo SIB.

Figure 11, Figure 12, Figure 13 and Figure 14 show the alpine meadow ground echo AIB, RIB, CIB, and SIB when the grazing angles are 20°, 45°, 65°, and 85°. It can be seen directly from the figures that the different grazing angle signals characteristic have obvious differences in figures. The AIB, RIB, CIB, and SIB are taken as signal features and as DBN input information. The figure abscissa is the number of points. The figure ordinate is normalized value. After the features are normalized, they are used as the network input. Normalization is conducive to the network training and prediction of the network, and avoids over-fitting and under-fitting.

Four grazing angles ground echo are classified with DBN. There are 60 sets of data for each grazing angle and 240 sets of data for four different grazing angles. The DBN classification result is shown in Table 5.

When the AIB is used as the input feature, the grazing angle 45° signal recognition rate reaches 98.33%, the grazing angle 65° signal recognition rate is low 51.67%, and the overall recognition rate reaches 78.33%.

When the CIB is used as the input feature, the four grazing angle recognition rates are basically consistent at approximately 70%. When the RIB is used as the input feature, the network has poor overall recognition rate. When the SIB is used as feature input, the 20°, 45°, and 65° recognition rates reach more than 80%, but the 85° signal recognition rate is very low at approximately 35%.

Through the above analysis, AIB as classification feature has the best overall recognition rate. According to the above analysis results, improve AIB. The AIB has no ability to suppress the trivial feature bispectrum points and interference bispectrum points. As a result, network uses the AIB feature as input and cannot complete the classification well. By introducing one-dimensional Fisher, important feature points are selected. Amplify important bispectrum features and suppresse trivial bispectrum points.

**Figure 15:**
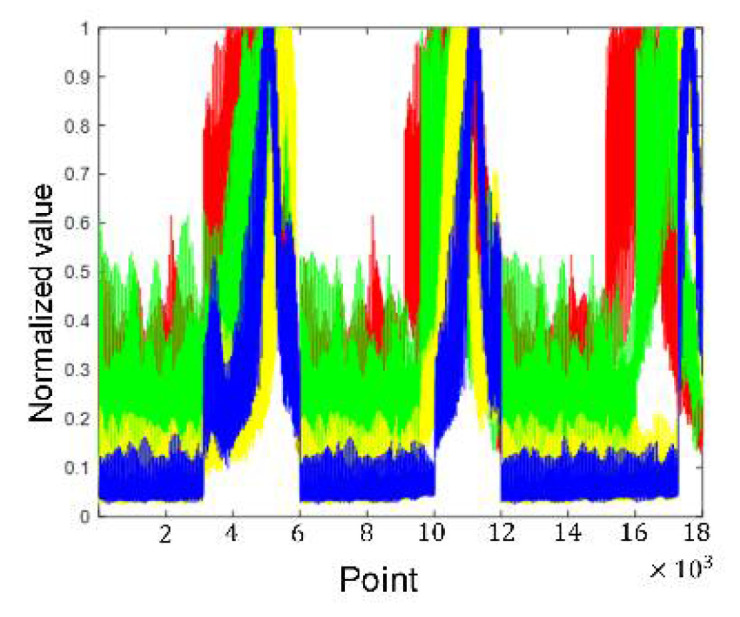
Selected AIB.

Figure 15 shows that the selected AIB amplifies the important points in the data and suppresses the trivial points. The DBN input vector of four grazing angles are very different, which is more conducive to the subsequent classification.

**Figure 16:**
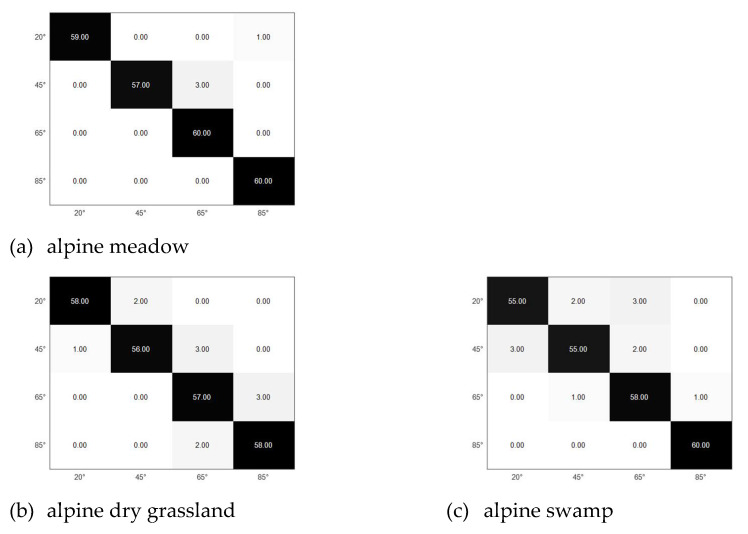
Selected AIB confusion matrix.

Use the selected AIB and DBN to classify the four grazing angles. The confusion matrix results are shown in Table 6 and Figure 16. 

The recognition rate of the four grazing angle signals can reach 95% in alpine meadow. The proposed selected AIB greatly increases the recognition rate of signals with different grazing angles. The processing method of network input data in this paper is of targets in typical alpine areas.

In order to verify the validity and versatility of the features, two different terrains, alpine dry grassland and alpine swamp, were selected for the grazing angle recognition. Figure 16 shows the confusion matrix of the two types of terrain angle recognition. Table 6 shows the result of the grazing angle recognition. Alpine dry grassland grazing angle recognition is more than 93%. Alpine swamp grazing angle recognition is more than 91%. It shows that the selected AIB feature is effective in identifying the glancing angle of typical alpine area targets.

## 6. Conclusions

In order to suppress the ground clutter of the terahertz radar, the statistical distribution characteristics of ground clutter amplitude under different grazing angles are analysed. Based on the bispectrum, the clutter selected AIB is extracted. Under the short data length, the characteristic information of the signal is retained. Clutter selected AIB is used as DBN input to complete grazing angle recognition. The study finds that the PDF curve of ground clutter amplitude is more in line with the normal distribution curve when the grazing angle is 0° ~ 45 °. The Weibull distribution and G0 distribution can describe the PDF curve of ground clutter amplitude at grazing angle 85°and 65°. The selected AIB can identify different grazing angle signals, and the recognition rate is greater than 95% in alpine meadow. The recognition rate of grazing angle is more than 93% in alpine dry grassland. The recognition rate of grazing angle is more than 91% in alpine swamp. It shows that the selected AIB feature is effective in identifying the glancing angle.

## Figures and Tables

**Table 1:** The 20° curve fitting error and $R^{2}$.

Distribution	Fitting Error	$R^{2}$
Rayleigh	0.69	0.41
K	0.74	0.34
Weibull	0.52	0.61
lognormal	0.35	0.82
Gamma	0.36	0.80
G0	0.18	0.95
K-root	0.33	0.83
normal	0.13	0.97

**Table 2:** The 45° curve fitting error and $R^{2}$.

Distribution	Fitting Error	$R^{2}$
Rayleigh	0.61	0.54
K	0.66	0.49
Weibull	0.33	0.83
Lognormal	0.18	0.95
Gamma	0.14	0.97
G0	0.13	0.97
K-root	0.15	0.97
normal	0.10	0.98

**Table 3:** The 65° curve fitting error and $R^{2}$.

Distribution	Fitting Error	$R^{2}$
Rayleigh	0.25	0.88
K	0.30	0.84
Weibull	0.14	0.96
Lognormal	0.22	0.91
Gamma	0.17	0.94
G0	0.14	0.96
K-root	0.18	0.93
normal	0.15	0.94

**Table 4:** The 85° curve fitting error and $R^{2}$.

Distribution	Fitting Error	$R^{2}$
Rayleigh	0.15	0.95
K	0.16	0.95
Weibull	0.12	0.96
Lognormal	0.20	0.91
Gamma	0.15	0.95
G0	0.12	0.97
K-root	0.17	0.93
normal	0.14	0.94

**Table 5:** The DBN classification result.

Distribution	20°	45°	65°	85°
AIB	78.33%	98.33%	51.67%	85.00%
CIB	76.67%	70.00%	66.67%	75.00%
RIB	73.33%	41.67%	65.00%	35.00%
SIB	85.00%	83.00%	88.33%	35.00%

**Table 6:** Selected AIB classification result.

	20°	45°	65°	85°
Alpine meadow	98.33%	95.00%	100%	100%
Alpine dry grassland	96.67%	93.33%	95%	96.67%
Alpine swamp	91.67%	91.67%	96.67%	100%

## Data Availability

The data presented in this study are available on request from the corresponding author. The data are not publicly available due to the database is being built. When the database is completed, it can be provided to researchers.
